# Soft Movable Polymer Gel for Controlling Water Coning of Horizontal Well in Offshore Heavy Oil Cold Production

**DOI:** 10.3390/gels8060352

**Published:** 2022-06-05

**Authors:** Jie Qu, Pan Wang, Qing You, Guang Zhao, Yongpeng Sun, Yifei Liu

**Affiliations:** 1 School of Energy Resources, China University of Geosciences, Beijing 100083, China; 2006210060@email.cugb.edu.cn (J.Q.); wangpanworkmail@gmail.com (P.W.); 2 School of Petroleum Engineering, China University of Petroleum (East China), Qingdao 266580, China; zhaoguang@upc.edu.cn (G.Z.); sunyongpeng@upc.edu.cn (Y.S.)

**Keywords:** polymer gel, water coning, chemical packer, horizontal well, offshore oil-field application

## Abstract

Horizontal well water coning in offshore fields is one of the most common causes of rapid declines in crude oil production and, even more critical, can lead to oil well shut down. The offshore Y oil field with a water cut of 94.7% urgently needs horizontal well water control. However, it is a challenge for polymer gels to meet the requirements of low-temperature (55 °C) gelation and mobility to control water in a wider range. This paper introduced a novel polymer gel cross-linked by hydrolyzed polyacrylamide and chromium acetate and phenolic resin for water coning control of a horizontal well. The detailed gelant formula and treatment method of water coning control for a horizontal well in an offshore field was established. The optimized gelant formula was 0.30~0.45% HPAM + 0.30~0.5% phenolic resin + 0.10~0.15% chromium acetate, with corresponding gelation time of 26~34 h at 55 °C. The results showed that this gel has a compact network structure and excellent creep property, and it can play an efficient water control role in the microscopic model. The thus-optimized gelants were successively injected with injection volumes of 500.0 m^3^. The displacement fluid was used to displace gelants into the lost zone away from the oil zone. Then, the formed gel can be worked as the chemical packer in the oil–water interface to control water coning after shutting in for 4 days of gelation. The oil-field monitoring data indicated that the oil rate increased from 9.2 m^3^/d to 20.0 m^3^/d, the average water cut decreased to 60~70% after treatment, and the cumulative oil production could obtain 1.035 × 10^4^ t instead of 3.9 × 10^3^ t.

## 1. Introduction

Waterflooding is the most economical and commonly used method for oil fields, especially for offshore oil fields with high equipment investment [1,2,3]. Due to the reservoir heterogeneity, it is inevitable that injected water will break into the oil well at the later stage of development, resulting in a high water production rate. Multi-branch, long-distance horizontal wells are the main way of drilling and production in offshore oil fields [4,5,6,7,8]. This is mainly due to the rational use of the operation space, saving resources, and increasing the oil drainage area of the reservoir. Once the formation water enters the horizontal well, the water cut increases sharply. A high level of water production leads to an increase in corrosion and scale, a high load of fluid-handling facilities, and significant environmental protection concerns, which eventually results in shut-in wells without economic benefit [9,10,11,12]. Therefore, efficient and low-cost water control methods are particularly important for the development of offshore oil fields.

Polymer gel treatment is a cost-effective method to improve sweep efficiency in reservoirs and reduce excess water produced during oil and gas production. Polymer gel treatment can also improve the rheology and composition of crude oil [13,14,15,16]. The gel formed by polymer and cross-linker can be injected into the target formation with high water cut and fully or partially seal the formation at reservoir temperature [17,18,19,20]. Polymer gels prepared with chromium acetate or phenolic resin are commonly used and relatively stable systems, but their gelation temperatures are very different [21,22,23,24]. Polymer gels prepared with chromium acetate or phenolic resin are commonly used and relatively stable systems, but their gelation temperature is very different. The activation temperature of phenol/formaldehyde cross-linked by acrylamide-based copolymer is above 80 °C, which is suitable for use in middle-/high-temperature oil reservoirs. When the reservoir temperature is low, the cross-linking condensation reaction of this system will slow down, and better formation effects cannot be guaranteed [25,26]. Since the formation of brine will affect the gelation time and stability of the dynamic gel, the researchers tried to develop a salt-insensitive gel system using a special salt-insensitive modified polymer (PAN, PVA, PAMPS, PSSA, etc.) [27]. Metal-based cross-linking agents (Cr^3+^, Al^3+^) are widely used in low-temperature reservoirs due to their low price, but the gelation time will decrease rapidly with the increase in gel temperature, which will lead to the problem of an uneven density of cross-links and poor gel stability. Organometallic cross-linkers can increase gelation time by slow release of metal ions [28,29]. Although the gel reaction rate can be controlled to some extent, the gel formed by metal complexation has poor toughness. It is more susceptible to damage during formation migration [30]. Moreover, the polymer gel should have good deformability, in addition to meeting the conditions of gel strength and gelation time, which easily leads to deeply migrating into the water-yielding formation and plugging water channels for enhanced oil recovery. The low-temperature composite cross-linked soft movable polymer gel can be cross-linked multiple times after being destroyed during the migration process. This gel with excellent strength and creep properties is more suitable for controlling water coning of horizontal wells in offshore oil fields.

The Y oil field is an offshore heavy oil field that has been put into scale development. It is a Neogene marine sandstone reservoir. HX well is situated in the formation NgⅡ_11+2_. For the formation NgⅡ_11+2_, the reservoir temperature is 55 °C, the reservoir pressure is about 13.5 MPa, the average porosity is 30.0%, the effective permeability is 2.1 μm^2^, and the salinity of formation water is 34,178.2 mg/L. The effective thickness of the sandstone reservoir is about 8.4 m. There is a water layer with sufficient bottom water energy under the production layer (NgⅡ_11+2_), resulting in a sharp rise in the water content of the production well. Recently, the water cut of the HX well has been up to 100%. When the bottom water breaks through, the great adverse mobility ratio of water to oil also promotes a sharp increase in water production and a significant decrease in oil production. In order to control water production, most horizontal wells need to take measures to block water channels and increase oil production. In this study, a novel soft movable polymer gel was introduced for controlling water coning of horizontal well in offshore heavy oil cold production. The strength and creep properties of gel were investigated, and its rheology and micromorphology are evaluated. Additionally, the treatment strategy was simulated and verified through a microscopic visual model. The oil-field application was implemented using the optimized treatment, and finally, good application results were achieved.

## 2. Experimental

### 2.1. Experimental Materials

Partially hydrolyzed polyacrylamide (HPAM, *M_w_*: 1.2 × 10^7^ Da, degree of hydrolysis: ~23%), chromium acetate (Cr^3+^) acetate cross-linker, and phenolic resin (PR, *M_w_*: 2 × 10^4^~3 × 10^4^ Da, sulfonation degree: ~20%) cross-linker were purchased from Shida Oil Field Service Company, Dongying, China. All samples were industrial grade (~95%). Y oil field HX well of reservoir temperature is 55 °C, and the formation water composition is shown in Table 1.

### 2.2. Preparation of Brine

High-density brine is an indispensable working fluid, which is usually determined by the density of the formation water. High-density brine is formulated by dissolving soluble inorganic salts. High-density brine used in oil-field construction usually has the following characteristics: (1) the density of brine is higher than that of the gelants; (2) the brine and formation water are compatible, and the viscosity is higher than that of the formation water; (3) the inorganic salts for preparing brine are cheap and environmentally friendly. Therefore, sodium chloride (NaCl) is an ideal water-soluble inorganic salt to prepare a high-density brine.

**Figure 1:**
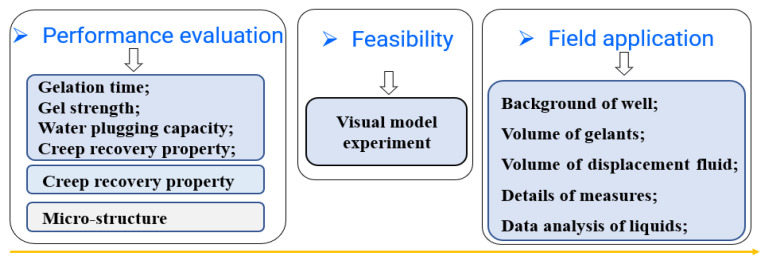
Flowchart of methodology.

Gel and brine were prepared as the basis of the material, and then the performance of the gel was systematically studied. The flowchart of methodology is shown in Figure 1.

### 2.3. Optimum Formula of Soft Movable Polymer Gels

(1) Gelation time

The polymer (0.3~0.45%) and cross-linker (0.3~0.6% PR + 0.05~0.20% Cr) were dissolved in tap water, and the pH value of the cross-linked glue solution is 6~6.5. Then, the mixed solution was placed in an oven at a temperature of 55 °C until the gel was completely gelled. Temperature triggered chromium ion release forms complex with a polymer carboxyl group, and phenolic resin is cross-linked with an amide group. Gelation time is the time when the gelants change from solution into code E according to the macroscopic visual inspection method (Sydansk’s method) commonly used in the oil field [31,32], as shown in Table 2. The gel strength was characterized by the code from A to G.

(2) Water-plugging capacity

**Figure 2:**
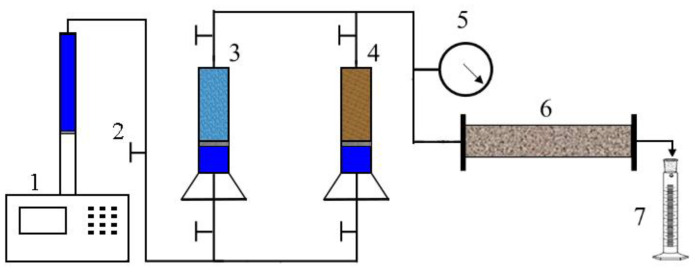
Schematic of the experimental set-up: 1—pump, 2—valve, 3—brine water container, 4—treatment fluid contianer, 5—pressure meter, 6—sand pack, and 7—graduated cylinder.

After the gelants enter the core pores, the polymer gels gelled by gelants can block the channeling channel. The water-plugging capacity was evaluated by comparing the permeability of the core after injection of gelants with the permeability of the original core. The schematic of the experimental setup is shown in Figure 2. The experimental procedures are as follows: (1) a sandbag was filled with dry sand as a reservoir unit, and its weight was measured; (2) the sandbag was evacuated overnight, saturated with water, and weighed. The volume of water (pore volume) was calculated from the mass of incoming water; (3) waterflooding was conducted, and the stable pressure was recorded; (4) 0.3 pore volume (PV) gelants were injected into a sand pack and placed into an oven, with reservoir temperature for a certain time; (5) waterflooding was implemented again, and the stable pressure was recorded; (6) the water-plugging capacity (*C*) and the residual resistance factor (*F*_rr_) of polymer gel were calculated, using Equations (1) and (2) as follows:

(1) $$C=(1-k_{b}/k_{a})\times 100\%$$

(2) $$\;F_{rr}=k_{b}/k_{a}=\Delta P_{a}/\Delta P_{b}\;$$

where *k*_b_—permeability of the sandbag before plugging; *k*_a_—permeability of the sandbag after plugging.

### 2.4. Creep Recovery Property

The creep and recovery properties of elastic polymer gels were measured using an MCR302 rheometer (Annton Parr Company, Germany), with a plate–plate at 55 °C. Constant shear stress of 2.4 Pa was applied on polymer gel, and the linear viscoelastic range was obtained. The strain of elastic polymer gel versus time was studied after eliminating the constant shear stress.

### 2.5. Microstructure of Gel

The gel microstructure was observed via environmental Quanta 200 FEG scanning electron microscopy (ESEM) (FEI Company, Hillsboro, OR, USA). During the experiments, a small piece of gel sample was directly placed on a covered ESEM grid at 25 °C.

### 2.6. Visual Physical Simulation Experiments

**Figure 3:**
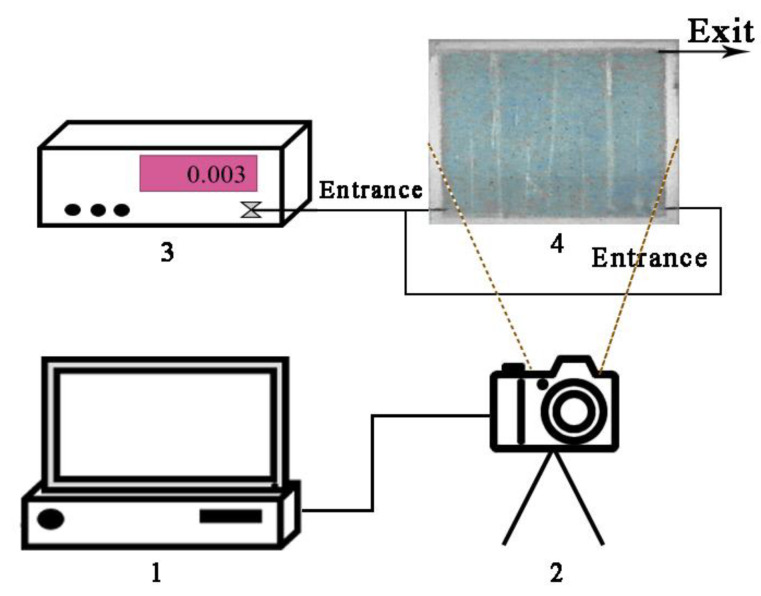
Schematic of visual physical simulation model: 1—computer, 2—video camera system, 3—syringe pump, and 4—model.

The visual physical model system was designed and built, as shown in Figure 3. The glass plate model consisted of an etched base glass plate combined with a cover glass plate, the boundaries of which were sealed with sealant. There were three ports at the bottom of the model—two water injection ports were located in the lower part, and one water outlet is located in the upper part. After the pumping device and the observation device were connected, the airtightness of the pipeline was checked, and the experiment of bottom-water coning and control in a horizontal well was simulated.

### 2.7. Flowchart of Soft Movable Polymer Gel for Controlling Water Coning in Horizontal Well

**Figure 4:**
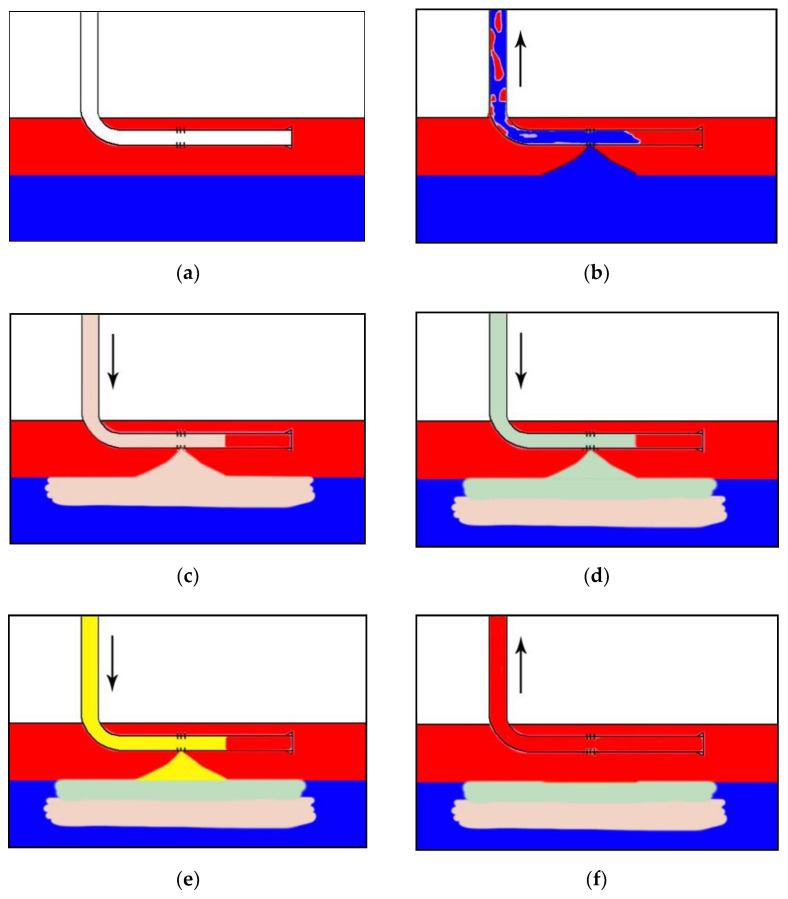
Schematic of polymer gel for controlling bottom-water coning in horizontal well: (**a**) reservoir with bottom water; (**b**) bottom water coning; (**c**) injecting high-density brine; (**d**) injecting the gelants; (**e**) injecting displacement fluid; (**f**) restarting well production: 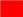 oil layer; 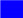 aquifer; 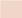 high-density brine; 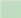 gelant; 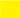 displacement fluid.

As shown in Figure 4, the horizontal well was located in the middle of the oil layer on a reservoir, and bottom water broke through and entered the wellbore from bottom to top (Figure 4a,b). Gravity separation is a core principle of controlling bottom-water coning technology, which relies on the density difference between fluids (oil and water) to cause gravity separation in the reservoir. In this study, first, high-density brine (0.99~1.02 g/cm^3^) was injected into the reservoir through the wellbore, and it mainly migrated to the water–oil transition zone to protect the polymer gel from entering the bottom of the water layer (Figure 4c). In the second step, the gelants with a density (0.97~0.98 g/cm^3^) between oil and brine were injected into the penetration zone (Figure 4d). Then, displacement fluid (~1.00 g/cm^3^) was injected to displace the gelants in the wellbore into the water coning zone and away from the oil layer of the reservoir (Figure 4e). Finally, after the displacement fluid was flushed away with fresh water from the mine, the horizontal well was shut in for gelation, completing a chemical gel packer (Figure 4f).

### 2.8. Construction Plan of HX Well

The water and oil production data of oil wells in recent years were first analyzed, after which the gel water control plan was determined. After calculating the usage of the three liquids, three working fluids had to be injected in sequence, mainly including high-density brine, gelants, and displacement fluid. The high-density brine was first injected into the bottom water layer to increase the density difference between the oil and water phases.

The gelants of polymer gel were injected into the bottom water layer through the water-cone water channel to act as a plug. The displacement fluid can drive the gelants of the wellbore and oil layer into the water layer to keep the oil flow channel unobstructed. After the injection of the three kinds of liquids, the HX well was shut in, which transformed the gelling agent form into a chemical gel packer near the interface between the oil and water layers, blocking the bottom-water coning. Finally, the data of water and oil production of oil wells after adopting gel water control were analyzed.

## 3. Results and Discussion

### 3.1. Density and Viscosity Properties of Brine

**Figure 5:**
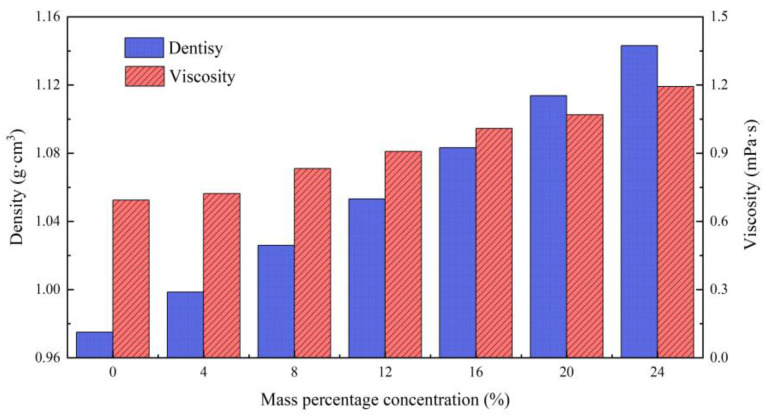
Density and viscosity of brine as a function of salt mass fraction at 55 °C.

The density and viscosity of brine as a function of salt mass fraction were measured, as shown in Figure 5. The density of the brine should be higher than that of the formation water in Table 1, so the mass fraction of sodium chloride in the preparation of high-density brine should be higher than 8%. Furthermore, there are two factors to consider. On the one hand, high-density brine may have a negative impact on the gelation of the gelants; on the other hand, the density between the high-density saline and transition zone also decreases exponentially during high-density saline and gel injection. The calculation found that when the injected high-density saline was 8% NaCl solution, the transition-density saline was half of that in high-density saline.

### 3.2. Optimum Formula of Soft Movable Polymer Gels

**Figure 6:**
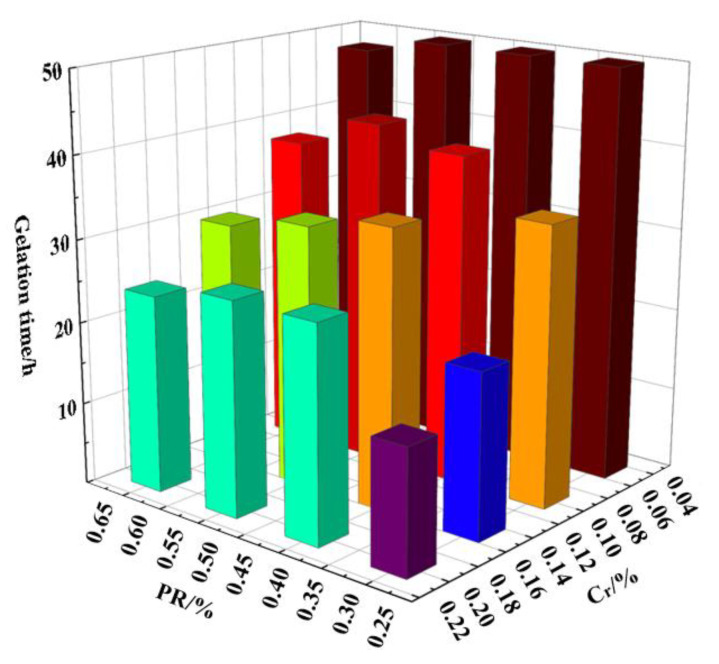
Histogram of gelation time (h) of 0.30% HPAM + PR and Cr^3+^ cross-linker.(The color represents gelation time of gel).

**Figure 7:**
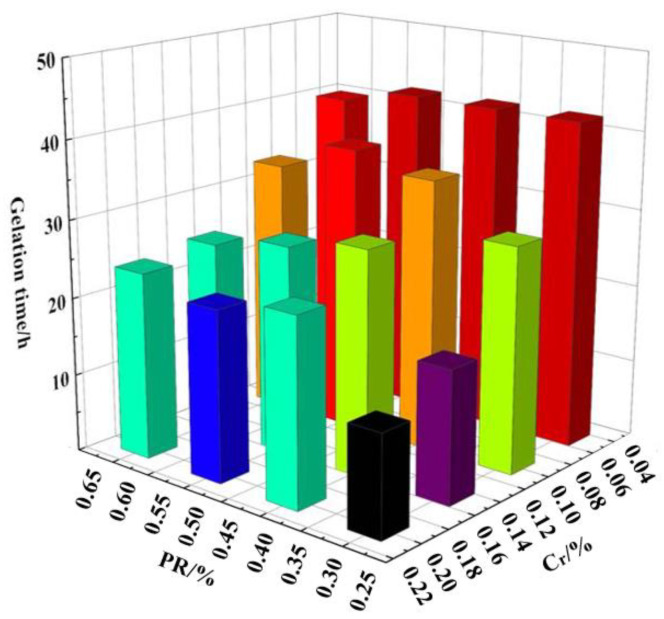
Histogram of gelation time (h) of 0.45% HPAM + PR and Cr^3+^ cross-linker. (The color represents gelation time of gel).

Based on temperature (55 °C) and salinity conditions (34,178.2 mg/L) of the Y oil-field reservoir, a novel polymer gel prepared via HPAM + PR and Cr^3+^ cross-linker was investigated. The gelation time, gel strength, thermal stability, and water-plugging capacity of the soft movable polymer gels were tested, as shown in Figure 6 and Figure 7, and Table 3 and Table 4.

The experimental results showed that the gelation time of gel prepared using HPAM + PR and Cr^3+^ cross-linker was 15~50 h. Additionally, the gel strength of most gels was higher than code F. After aging for 30 days at 55 °C, the gel stability of these gels slightly decreased, and the thermal stability of gel strength was very good.

**Figure 8:**
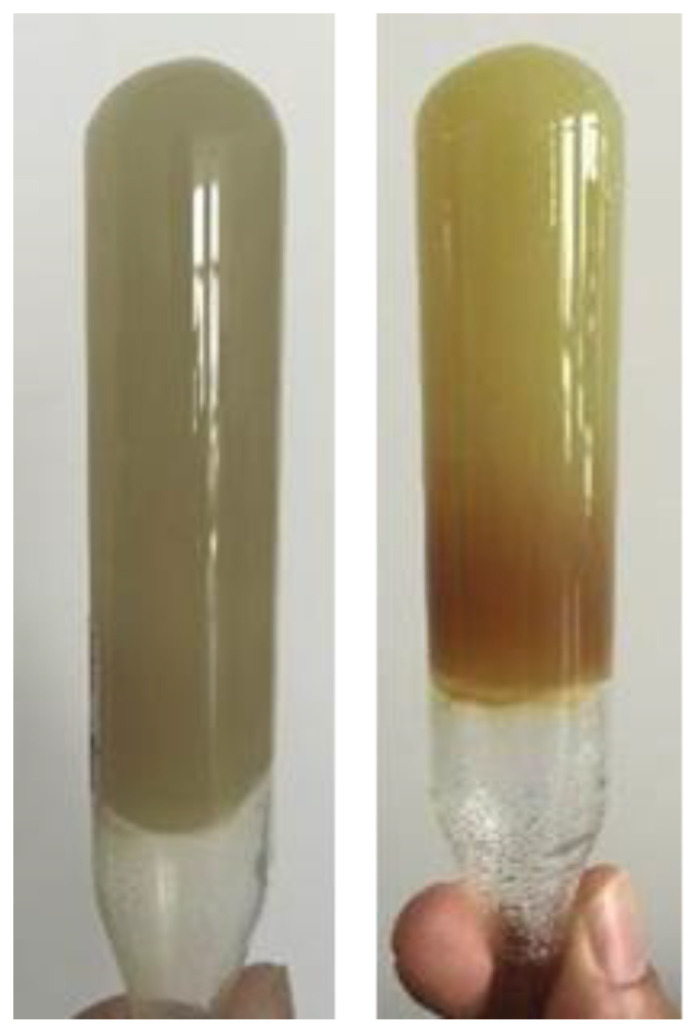
The gelation performance of gel prepared via PR and Cr^3+^ cross-linker after aging 3 d and 30 d, respectively.

It is necessary to guarantee safe injectivity into the horizontal well by using gelants with a long gelation time. Both the near- and far-well formulas of gels must satisfy the gelation time of greater than 24 h for construction. The residual resistance factor of the gel used in the far well should not be too large (*F*_rr_ < 50), and the residual resistance factor of the gel used in the near well should be higher than that of the far well (*F*_rr_ < 200). According to practical experience and economic benefits, two kinds of formulas of polymer gels were selected: 0.30% HPAM + 0.3% PR and 0.1% Cr^3+^ cross-linker, as well as 0.45% HPAM + 0.5% PR and 0.15% Cr^3+^ cross-linker. Taking one formula of the polymer gel as an example, Figure 8 shows the gelation performance of the gel prepared via 0.30% HPAM + 0.30% PR and 0.1% Cr^3+^ after aging 3 days and 30 days. It can be seen that the gel has no syneresis even after aging for 30 days.

It can be seen from Table 5 that plugging efficiency was more than 98% for different formulas of gel systems. Moreover, with the increase in polymer and cross-linker concentration, both the plugging efficiency and residual resistance factor were improved. This indicates that the gel system has good plugging properties.

### 3.3. Creep Recovery Property

**Figure 9:**
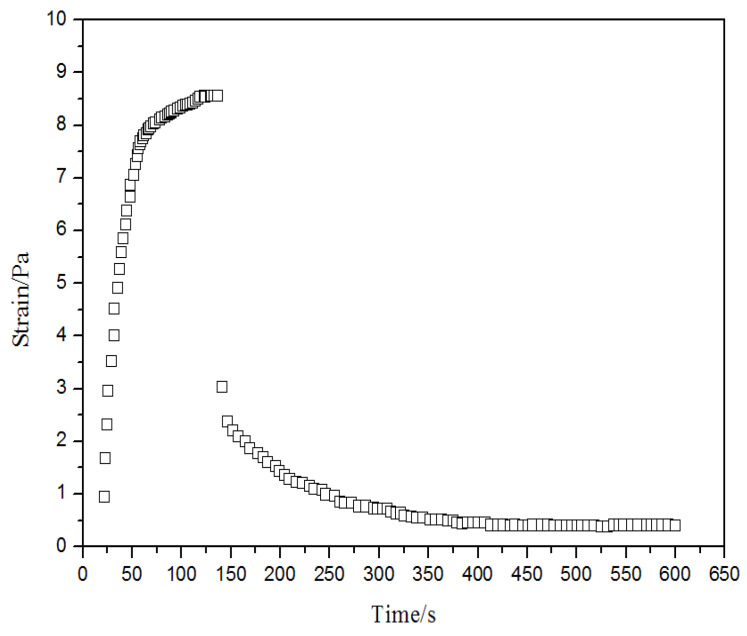
Elastic characteristics of the gel system at 55 °C.

The creep recovery property of the gel is shown in Figure 9. The polymer gel good deformability, which easily leads to deep migration into water-yielding formation and water plugging for enhanced oil recovery. By analyzing the strain characteristics of the gel, the creep recovery process can be divided into two phases. In the first phase, the constant stress (2.4 Pa) acted on the gel samples for 120 s. The initial strain increased sharply and then tended to become stable over time. The connections between the main structural units in the elastic gels were stretched elastically, and the strain value was several times than that of initial state at the constant shear stress. In the second phase, the constant stress was removed from the gel samples, and the strain could be recovered. The metallic chromium cross-linked gel destroyed its viscoelasticity under shear stress. It can be concluded the deformation and recovery abilities of the gel prepared via HPAM + PR and Cr^3+^ cross-linker is strong, which means that the gel has strong deformability and good elasticity.

### 3.4. Microstructure of the Gel Systems

**Figure 10:**
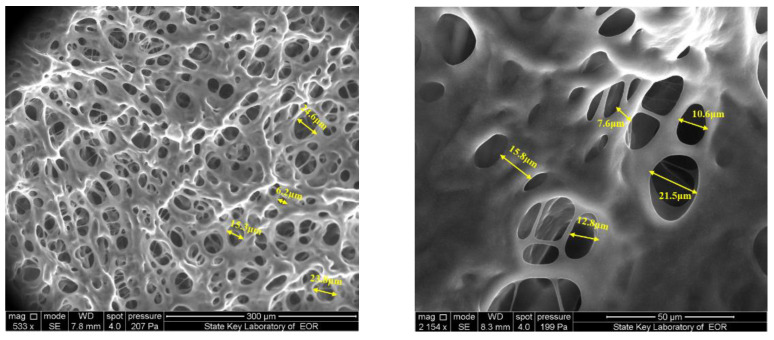
ESEM micrographs of the gel sample.

ESEM is a good way to accurately investigate the microstructure of the gel system. This method can keep the gel system from damage and observe the gel samples in their natural state. The ESEM micrograph of the gel sample is shown in Figure 10. It can be noted that the gel sample had a uniform three-dimensional network. Additionally, the pore size ranged from 5.0 μm to 28.0 μm, and the border thickness changed from 10.0 μm to 30.0 μm for the gel sample. A single chromium metal cross-linking agent forms a large network hole and a thin omentum [25,26], which is easy to dehydrate under external force damage. It can be concluded that the composite cross-linked gel has a more compact structure than that of metal cross-linked gels, which contributes to the good stability of the gel system.

### 3.5. Visual Model Experiment of Water-Coning Control in the Horizontal Well

**Figure 11:**
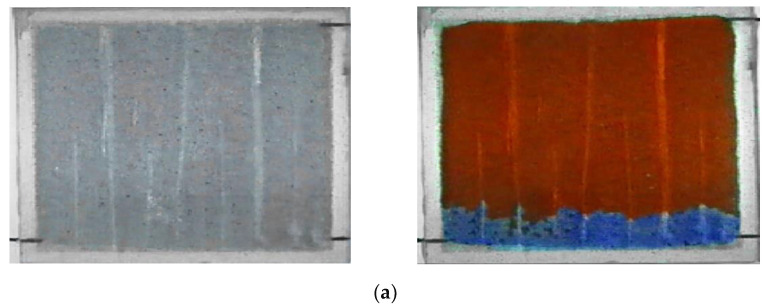

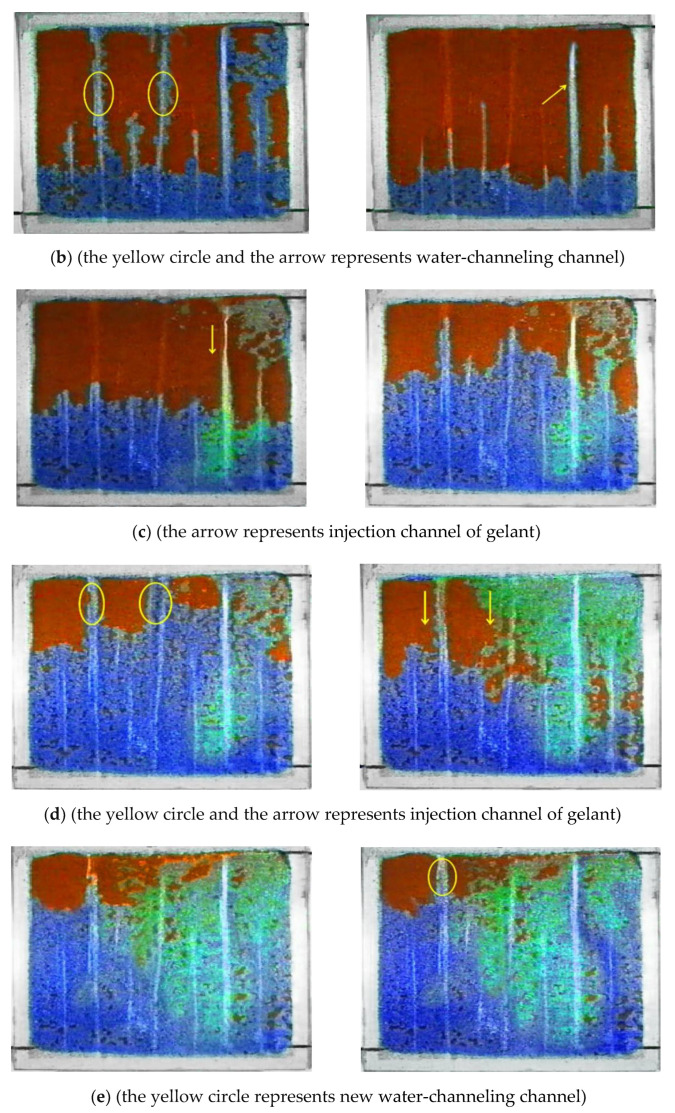
Visual physical simulation experiments: (**a**) horizontal well with bottom water; (**b**) bottom water coning; (**c**) injecting high-density brine and transition-density brine and injecting the gelants; (**d**) injecting displacement fluid; (**e**) resumption of production.

As shown in Figure 11, the reservoir model with bottom water was placed vertically, and the middle point at top of the model was used to simulate the water coning point of the horizontal well. The vertical stripe was the water-channeling channel in the matrix model. The simulated oil (0.89 g/cm^3^, ~180 mPa·s) was injected from the water injection port at the bottom of the model to saturate the model, and then the water was injected after the crude oil aged and stabilized (Figure 11a). The simulated oil was produced from the top outlet in the model to mimic oil production from the horizontal well with bottom water (Figure 11b). As oil was produced from the top of the model, bottom-water coning would occur (Figure 11b). High-density brine has a higher density than plugging agent and higher viscosity than formation water, so it entered the bottom aquifer along the water coning channels. The brine had little effect on the performance of the following gel. The transitional density saline was injected into the well when the high-density saline was completed, and it separated the high-density saline and the polymer gel and weakened the effect of salt on gelant properties. The gelants entered the bottom aquifer along the water-coning channels and spread in a horizontal direction during their injection stage (Figure 11c). The displacement fluid displaced gelants in the vicinity of the oil–water interface (Figure 11d). Then, the top production port was shut in order to wait for the transformation of gelants into gels. The chemical gel packer can effectively solve horizontal bottom-water coning and increase oil production (Figure 11e).

From the experimental results in the above figure, it can be seen that the injection of high-density brine increased the density difference between the bottom water and oil. The gelants’ density ranged from 0.97 g/cm^3^ to 0.98 g/cm^3^, which is between high-density brine (ρ8%NaCl = 1.0255 g/cm^3^) and oil (ρ_o_ = 0.886 g/cm^3^). Thus, this chemical gel packer formed near the oil–water transition layer. When the chemical gel packer was completed to control bottom-water coning, it was difficult for the bottom water to flow into the horizontal well. The longer the gel packer stability was, the better the effect of controlling bottom-water coning was.

### 3.6. Field Application

#### 3.6.1. Background of HX Well

**Figure 12:**
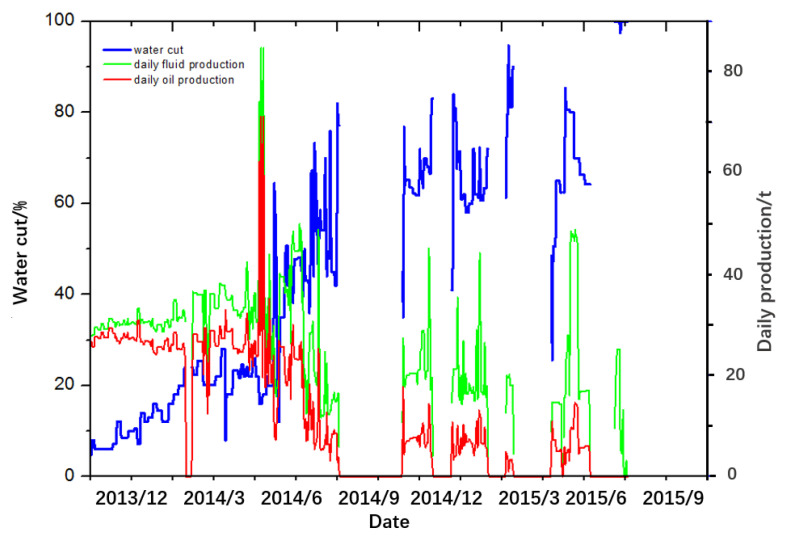
Data of crude oil and liquids production over time in HX well before treatment.

The pay zone of the Y oil field is NgⅡ_11+2_, with a formation thickness of 8.4 m and a reservoir temperature of 55 °C. The depth of the HX well is 2045.0 m, and the horizontal section length is 382.0 m. The data of crude oil and liquids production in the HX well over time are presented in Figure 12. Due to a high water cut (>90%) in Mar 2015, measures were taken in the HX well to control excess water production using polymer gel in March 2016.

The main reasons for choosing a chemical gel packer are as follows: (1) The oil layer is thin (<10.0 m), and the horizontal well section is located lower and close to the oil–water interface; (2) there is no tight layer at the bottom of the oil layer to block bottom water; (3) the serious vertical heterogeneity of the reservoir is conducive to the gravity separation of injected fluid.

#### 3.6.2. Gelants Formula (A + B) of Polymer Gel

(1) Gelant A formula for far from wellbore

Gelant A were made up of 0.30% HPAM + 0.30% PR and 0.10% Cr^3+^ cross-linker, and the gelation time was 36 h at 55 °C;

(2) Gelant B formula for near-wellbore zone

Gelant B were made up of 0.45% HPAM + 0.50% PR and 0.15% Cr^3+^ cross-linker, and the gelation time was 24 h at 55 °C.

#### 3.6.3. Volume of Gelants

Equation (3) for calculating the volume of high-density brine is shown as follows:

*V*_1_ = 2 × (*R*_1_ − *R*_2_) × *L* × *φ* × *β* (3)

where *V*_1_—the volume of high-density brine, m^3^; *R*_1_—radius of the brine layer, m; *R*_2_—radius of gelants layer, m; *L*—length of horizontal segment, m; *φ*—porosity, %; *β*—the ratio of the width of vertical high permeability to the length of horizontal segment [18].

For HX well, *L* = 382.0 m, *φ* = 30.4%. Assuming *R*_1_ = 12.0 m, *R*_2_ = 11.0 m and *β* = 20%, then volume of high-density brine as follows:

*V*_1_ = 2 × (12 − 11) × 382 × 30.4% × 20% = 47.0 m^3^

The designed *V*_1_ was 50 m^3^. The volume ratio of the high-density brine (8% NaCl) to transition density brine (4% NaCl) was 1:1, so each volume of them was 25.0 m^3^.

Equation (4) for calculating the volume of polymer gel is shown as follows:

*V_2_* = 2 × (*R*_2_ − *R*_3_) × *L* × *H* × *β* × *φ* (4)

where *V*_2_—the volume of polymer gel, m^3^; *R*_2_—radius of gelants, m; *R*_3_—radius of displacement fluid, m; *L*—length of horizontal section, m; *φ*—porosity, %; *β*_1_—the proportion of fluid channeling (≤5%).

If *L* = 382.0 m, *φ* = 30.4%, *R*_2_ = 11.0 m, *R*_3_ = 3.0 m and *β*_1_ = 3.1%, then volume of polymer gel as follows:

*V*_2_ = 2 × (11 − 3) × 382 × 8.4 × 30.4% × 3.1% = 500.0 m^3^

The designed *V*_2_ was 500.0 m^3^. The two gelants were divided into two parts according to the volume ratio of 3:2 (A:B), and the volume of gelant A for far from wellbore was 500.0 × 3/5 = 300.0 m^3^, while the volume of gelant B for the transition zone was 500 × 2/5 = 200.0 m^3^.

Equation (5) for calculating the volume of displacement fluid is shown as follows:

*V*_3_ = 2 × *R*_3_ × *L* × *H* × *φ* × *K* (5)

where *V*_3_—volume of displacement fluid, m^3^. Then, volume of displacement fluid as follows:

*V*_3_ = 2 × 3 × 382 × 8.4 × 30.4% × 3.1% = 181.0 m^3^

The gelants’ density of the above gel formula was between 0.97 g/cm^3^ and 0.98 g/cm^3^, which is between high-density brine (*ρ*_8%NaCl_ = 1.0255 g/cm^3^) and oil (*ρ*_o_ = 0.886 g/cm^3^). Therefore, the chemical packer formed by gel was easily built up near the oil–water interface.

#### 3.6.4. Detail of Measurements

(1) The HX well maintained production for 10 days and restored the existing water cone channel; (2) briefly, 50.0 m^3^ high-density brine was injected into the HX well; (3) then, 300.0 m^3^ far-wellbore gelant was injected, after which 200.0 m^3^ near-wellbore gelant was injected into HX well; (4) then, 181.0 m^3^ displacement fluid was injected into HX well; (5) seawater was used as a displacement fluid to drive gelants into percolation water layer; (6) the HX well was shut in for 4 days, and the gelants became a chemical gel packer; (7) the HX well was reopened, and production resumed.

#### 3.6.5. Field-Test Results

**Figure 13:**
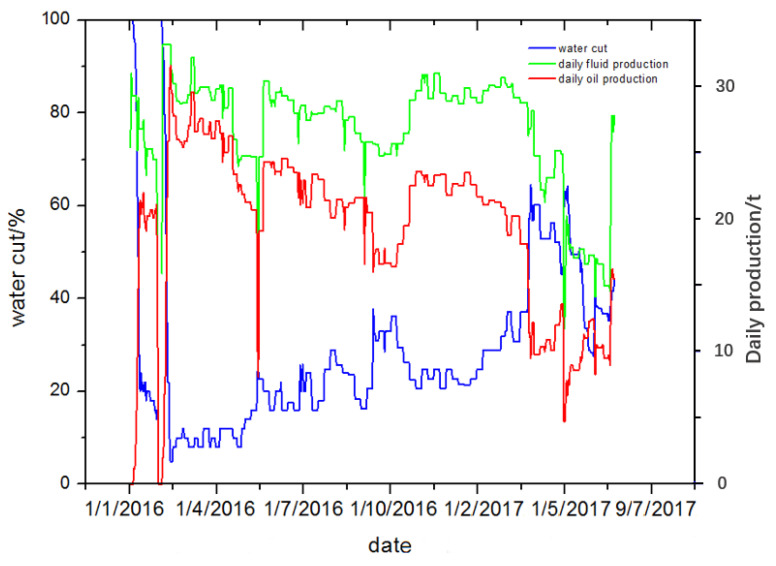
Data of crude oil and liquids production over time in HX well after treatment.

The data of crude oil and liquids production over time in HX well after treatment is shown in Figure 13. It can be seen that the highest total oil production per day of the HX well was 29.6 t, and the water cut decreased to 8% and remained at a lower level, less than 50% for a long time after the treatment in the HX well. Lastly, the accumulated oil production was 1.035 × 10^4^ t instead of 3.9 × 10^3^ t. Oil-field construction results confirmed that this novel method is quite suitable for water coning control and has a broad application prospect.

## 4. Conclusions

(1) The designed gelant formula of soft movable polymer gel was 0.30~0.45% HPAM + 0.30~0.5% phenolic resin + 0.10~0.15% chromium acetate, with a corresponding gelation time of 26~34 h at 55 °C. The formula meets the technological requirements of controlling bottom-water coning in horizontal wells in offshore oil fields.
(2) The visual glass plate simulation device can intuitively present the phenomenon of bottom-water coning and the effect of high-density brine injection and chemical packer control, which confirmed the feasibility of the process.
(3) Combined with the production status and reservoir conditions of the HX well in the Y oil field, the technology of soft movable polymer gel for controlling water coning was successfully applied, which provides a technical basis for cost reduction and efficiency improvement in offshore oil fields.

## Figures and Tables

**Table 1:** Composition of Y oil-field formation water.

Components	Na^+^ and K^+^	Ca^2+^	Mg^2+^	HCO_3_^−^	SO_4_^2^^−^	Cl^−^
Concentration (mg/L)	10,516.2	423.5	1524.6	139.8	2821.6	18,752.5

**Table 2:** Gel strength ratings and descriptions [17].

Code	Detailed Gel Description
A	**No detectable gel formed:** gel has the same viscosity as the original polymer solution.
B	**Highly flowing gel:** gel is slightly more viscous than the original polymer solution.
C	**Flowing gel:** most of the gel flows to the vial/tube top upon inversion.
D	**Moderately flowing gel:** only a small portion (5~15%) of the gel does not readily flow to the vial/tube top upon inversion.
E	**Barely flowing gel:** gel barely flows to the vial/tube top; a significant portion (>15%) of the gel does not flow upon inversion.
F	**Highly deformable nonflowing gel:** gel does not quite reach the vial/tube top upon inversion.
G	**Moderately deformable nonflowing gel:** gel deforms about half the distance to the vial/tube top upon inversion.
H	**Slightly deformable nonflowing gel:** gel surface slightly deforms upon inversion.
I	**Rigid gel:** no gel surface deformation occurs upon inversion.
J	**Ringing rigid gel:** a tuning fork-like mechanical vibration occurs upon tapping.

**Table 3:** Gel strength and long-term thermal stability of the gel system prepared via 0.30% HPAM + PR and Cr^3+^ cross-linker.

HPAM/%	PR/%	Cr^3+^/%	Gel Strength/%	Aging 30 d at 55 °C
0.30	0.30	0.05	D~E	No syneresis
		0.10	F	No syneresis
		0.15	E	No syneresis
		0.2	E	No syneresis
	0.40	0.05	C~D	No syneresis
		0.10	F	No syneresis
		0.15	F	No syneresis
		0.20	E~F	No syneresis
	0.50	0.05	C	No syneresis
		0.10	F	No syneresis
		0.15	F~G	No syneresis
		0.20	F	No syneresis
	0.60	0.05	C	No syneresis
		0.10	E~F	No syneresis
		0.15	F~G	No syneresis
		0.20	F~G	No syneresis

**Table 4:** Gel strength and long-term thermal stability of the gel system prepared via 0.45% HPAM + PR and Cr^3+^ cross-linker.

HPAM/%	PR/%	Cr^3+^/%	Gel Strength/%	Aging 30 d at 55 °C
0.45	0.30	0.05	F	No syneresis
		0.10	F	No syneresis
		0.15	F~G	No syneresis
		0.2	F~G	No syneresis
	0.40	0.05	D~E	No syneresis
		0.100	F~G	No syneresis
		0.15	F~G	No syneresis
		0.20	G	No syneresis
	0.50	0.05	C	No syneresis
		0.10	F	No syneresis
		0.15	G~H	No syneresis
		0.20	G~H	No syneresis
	0.60	0.05	C	No syneresis
		0.10	F	No syneresis
		0.15	G~H	No syneresis
		0.20	G~H	No syneresis

**Table 5:** Plugging capacity of the gel system.

Gel Type	Gel Formula (wt)	Gelation Time/h	Initial Permeability/μm^2^	Plugging Efficiency/%	Residual Resistance Factor
HPAM + PR + Cr^3+^	0.30% + 0.50% + 0.20%	26	3.89	99.85	769.2
	0.30% + 0.40% + 0.15%	34	3.09	99.16	109.1
	0.30% + 0.30% + 0.10%	32	4.24	96.94	32.2
	0.45% + 0.50% + 0.20%	22	3.87	99.65	356.8
	0.45% + 0.4% + 0.15%	24	3.08	98.75	80.0
	0.45% + 0.3% + 0.10%	24	2.83	95.75	23.5

## Data Availability

Data are contained within the article.
